# Process Evaluation of “The Hygienic Family” Intervention: A Community-Based Water, Sanitation, and Hygiene Project in Rural Malawi

**DOI:** 10.3390/ijerph19116771

**Published:** 2022-06-01

**Authors:** Mindy Panulo, Kondwani Chidziwisano, Tara K. Beattie, Elizabeth Tilley, Christabel Kambala, Tracy Morse

**Affiliations:** 1 Centre for Water, Sanitation, Health and Appropriate Technology Development (WASHTED), Malawi University of Business and Applied Sciences, Private Bag 303, Chichiri, Blantyre 3, Malawi; kchidziwisano@poly.ac.mw (K.C.); tracy.thomson@strath.ac.uk (T.M.); 2 Department of Environmental Health, Malawi University of Business and Applied Sciences, Private Bag 303, Chichiri, Blantyre 3, Malawi; ckambala@mubas.ac.mw; 3 Department of Civil and Environmental Engineering, University of Strathclyde, Level 5 James Weir Building, Glasgow G1 1XQ, UK; t.k.beattie@strath.ac.uk; 4 Department of Mechanical and Process Engineering, ETH Zurich, 8092 Zurich, Switzerland; tilleye@ethz.ch

**Keywords:** process evaluation, hygiene, WASH, Malawi, food safety, food hygiene, community health

## Abstract

Process evaluations of environmental health interventions are often under-reported and under-utilized in the development of future programs. The “Hygienic Family” intervention targeted improvements in hygiene behaviors of caregivers with under five-year-old children in rural Malawi. Delivered through a combination of open days, cluster meetings, household visits, and prompts, data were collected from two intervention areas for ten months. A process evaluation framework provided indicators that were measured through intervention implementation and expenditure reports, focus groups discussions, interviews, and household surveys. The collected data assessed the intervention fidelity, dose, reach, acceptability, impact, and cost. Results indicated that all planned hygiene promotion messages were delivered, and study participants were better reached primarily through household visits (78% attended over 75% of the intervention) than cluster meetings (57% attended over 75% of the intervention). However, regression found that the number of household visits or cluster meetings had no discernible effect on the presence of some household hygiene proxy indicators. Intervention implementation cost per household was USD 31.00. The intervention delivery model provided good fidelity, dose, and reach and could be used to strengthen the scope of child health and wellbeing content. The intensive face-to-face method has proven to be effective but would need to be adequately resourced through financial support for community coordinator remuneration.

## 1. Introduction

Diarrheal disease is the second leading cause of death in children under five years old, and more than half a million children under five die annually [1]. Despite improvements in diarrhea mortality rates, the disease still accounts for approximately 10% of all fatalities in children under the age of five and is responsible for half a million child deaths each year in low- and middle-income countries (LMICs) [2,3]. The current state of continued childhood diarrhea disease prevalence accompanied by unsuccessful water, sanitation, and hygiene (WASH) trials have led to a call for transformational approaches (i.e., a comprehensive package of interventions tailored to address local exposure landscapes and enteric disease burden) to promote WASH behaviors [4,5,6]. The transformational approaches should be effective in terms of health outcomes, acceptability, and scalability to improve child health in LMICs [7].

Behavior promotion strategies are all designed differently from each other in terms of their theoretical grounding, the content, delivery mode, and effectiveness [8,9,10]. Thus, knowing which approaches are worthy of future investment through conducting “Process Evaluations” is essential [7]. However, over time, more attention has been given to evaluating the outcome of the health promotion program rather than assessing the process by which the intervention was delivered [11], with inadequate reporting on how such programs are implemented through process evaluations [7,12,13,14]. Thus, there has been limited application and use of process evaluation processes to improve behavior change interventions. Therefore, we conducted a process evaluation of a theory-based, complementary food hygiene and WASH intervention (the “Hygienic Family”) in Malawi.

The “Hygienic Family” (Banja La Ukhondo) was a theory-based, complementary food hygiene and WASH intervention targeting behaviors of caregivers of children aged six months to two years, which was designed and implemented in rural Malawi [15]. Formative research indicated that norms, ability, and self-regulation factors were the primary determinants of selected behaviors. The intervention was delivered for 9 months (February–October 2018) through open days, cluster meetings, and household visits across two treatment groups. Treatment group 1 focused on two behavior packages, namely (1) handwashing with soap and (2) food safety and hygiene, while treatment group 2 targeted four key behaviors: (1) handwashing with soap, (2) food safety and hygiene, (3) feces management, and (4) water management (safe water management). The intervention was delivered by community coordinators and health surveillance assistants (HSAs), who were trained in both content and delivery mechanisms by treatment arm coordinators. These trainings were practical, hands-on, and competence-based. At end line, results showed a significant reduction in self-reported diarrhea and positive social outcomes [16,17], significant increases in the presence of proxy measures in treatment groups (e.g., the presence of soap), and a significant increase in three target behaviors: handwashing with soap, washing kitchen utensils with soap, and keeping kitchen utensils in a safe place [18]. Process evaluation of the intervention assessed the fidelity, dose, reach, acceptability, and cost of the “Hygienic Family” intervention. This paper presents the methodology and key findings of the Hygienic Family process evaluation, which highlights important areas that may be considered during intervention scale-up.

## 2. Materials and Methods

### 2.1. Study Setting

The study was conducted in the Chikwawa district, which is located in the southern region of Malawi. With a population of 564,684 [18], 12% of people use unsafe water sources for drinking, while 9% continue to practice open defecation [18]. At district level, the prevalence of diarrhea among children under five years old is 18% (DHS, 2016). The Hygienic Family intervention was implemented in 50 villages of three traditional authorities (TAs) of the Chikwawa district among 800 caregivers.

The intervention was designed based on the risks, attitudes, norms, abilities, and self-regulation (RANAS) behavior change model. The RANAS model is an approach to systematic behavior change and an established method for designing and evaluating behavior change strategies that target and change the behavioral factors of a specific behavior in a specific population [19].

**Figure 1:**
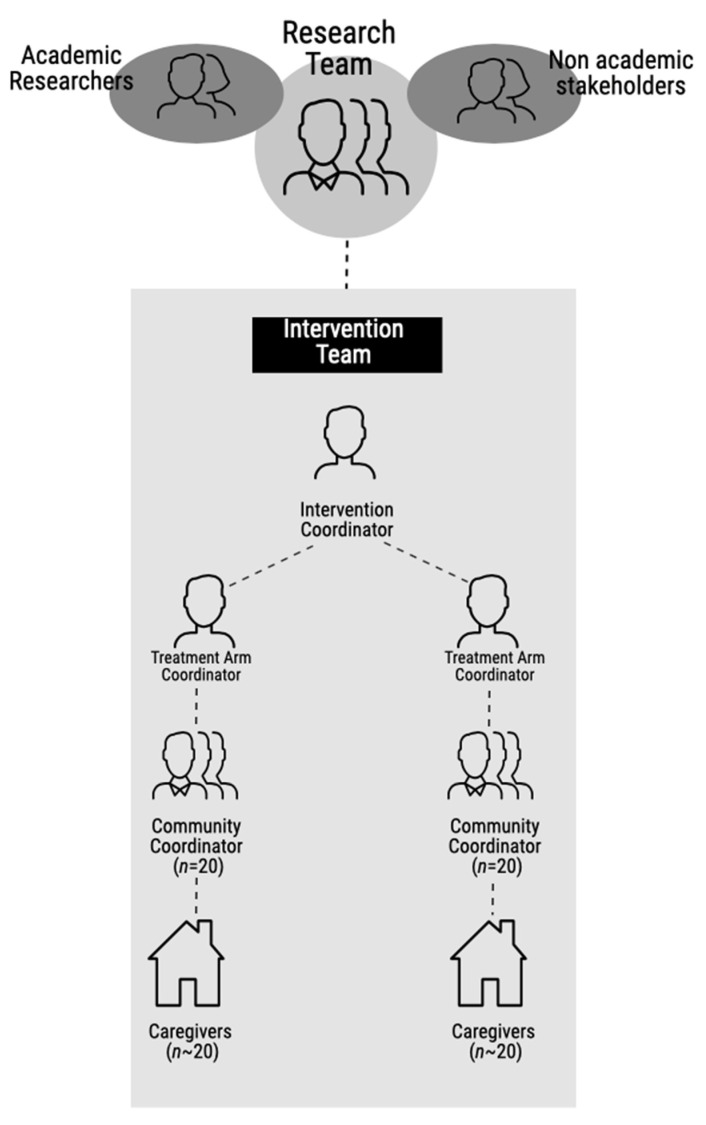
Outline of the Hygienic Family intervention implementation structure.

The Hygienic Family intervention worked with forty community coordinators (frontline intervention implementors) and two drama groups. Each community coordinator was responsible for a cluster of up to 25 caregivers (recipients of the intervention). Each treatment arm was supported and supervised by a treatment arm coordinator, who were in turn supported by an intervention coordinator, overseen by a dedicated research fellow and the principal investigator (Figure 1). Intervention implementation included the provision of items including paint, Glo Germ^TM^ (Moab, UT, USA), development of training manuals (printing, binding, and laminating of training manuals) and illustrations, PA systems (hired), consumables (e.g., paper plates, spoons, and fruits), and hygiene consumables, e.g., soap (Appendix A). Details of the trial design and methods are described elsewhere [15,17].

### 2.2. Evaluation Design and Framework

**Figure 2:**
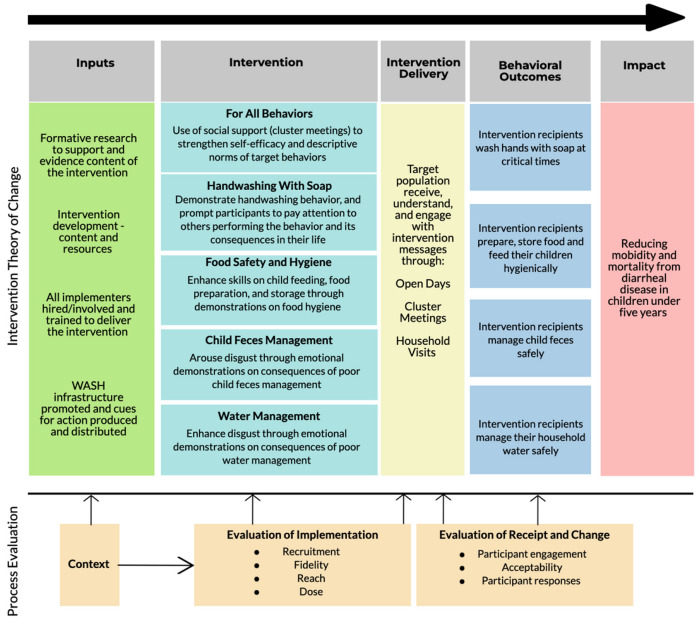
Process evaluation framework.

The process evaluation was guided by the medical research council (MRC) process evaluation framework [13,20]. The process evaluation focused on seven evaluation domains from the process evaluation framework by Linnan and Steckler [20]. Additionally, the cost of the intervention across the two treatment groups was evaluated. Figure 2 shows how the intervention operational elements were hypothesized to bring about change in the targeted behaviors with an ultimate goal of reducing diarrhea in children under five years old. The process evaluation domains are grouped into categories of “implementation”, “receipt and change mechanisms”, and “context”, and the timing of their measurement is shown in Table 1. The intervention delivery aspects were measured using the implementation associated domains, whilst receipt and mechanisms of change explored the effects of the delivered intervention content.

The constructs used in the process evaluation included:

- Fidelity: delivery of the intervention as intended;
- Dose delivered: the quantity of events actually implemented;
- Reach: the extent to which the intended audience participated in the intervention;
- Recruitment strategies: the techniques applied to mobilize intervention recipients;
- Participant engagement and responses: receipt and understanding of the key messages and interaction with the content;
- Acceptability: assessing the intervention acceptability of both the implementers and the recipients;
- Context: the environmental setting supporting or impeding intervention delivery, receipt, and uptake.

### 2.3. Data Collection

Using a mixed-methods approach (i.e., qualitative and quantitative) data were collected in the intervention population throughout the implementation period (February to October 2018) and at the end of the intervention (November 2018). The data included intervention implementation reports; focus group discussions (FGDs) with community health workers—also known as health surveillance assistants (HSAs)—community chiefs, caregivers, and community coordinators; in-depth interviews with implementers (community coordinators, treatment arm coordinators, and intervention coordinator); and a household questionnaire survey with each intervention household. Details of the data available for the process evaluation are shown in Table 1 and described below.

#### 2.3.1. Implementation Summary Reports and Weekly Activity Reports

Community coordinators wrote weekly reports during delivery of the intervention. The report included records of attendance, the availability of supplies used for the activities, and challenges, if any, that were encountered during intervention delivery. Attendance was captured through listing (names) of the attendees. Treatment arm coordinators compiled these weekly reports and produced an overview report at the completion of each package. The process evaluation accessed twenty-six weekly reports from treatment arm 1 and thirty-four weekly reports from treatment arm 2 (due to their longer intervention period).

Additionally, before community coordinators delivered each behavior package, they were trained by treatment arm and intervention coordinators. For these trainings, records of attendance, availability of materials for training, and challenges faced, if any, that may have affected the training were recorded. Such reports were then used to assess the fidelity, dose delivered, and reach achieved with the trainings.

#### 2.3.2. Key Informant Interviews

Using purposive sampling, the two-treatment arm and intervention coordinators (BSc Environmental Health/Public Health holders) were interviewed post intervention. Using an interview guide, the interviews aimed to understand these implementers perception and acceptability towards the intervention delivery method. Additionally, interviews focused on the successes and challenges encountered while delivering the intervention and strategies applied to attract the intervention recipients.

#### 2.3.3. Supervisory Visits

The intervention coordinator made unannounced visits intermittently throughout the intervention period to monitor randomly selected cluster meetings and household visits. Observations made during such supervisory visits were recorded in the form of a report (*n* = 6). The reports included details on the setting; fidelity, including competence of the community coordinators in delivering the intervention; and how the participants reacted to the activities. The coordinator also included details of any technical problems encountered while delivering the intervention and the attendance of the participants. As part of the supervisory visits, the intervention coordinator gave feedback to the community coordinators to improve the fidelity of the intervention.

#### 2.3.4. Focus Group Discussions

With the use of a focus group discussion (FGD) guide, FGDs were held with village chiefs (*n* = 2), HSAs (*n* = 2), and community coordinators (*n* = 2). For the various respondents (chiefs, HSAs, and community coordinators), one FGD represented treatment arm 1 and 2, respectively. These FGDs focused on understanding the wider acceptability of the intervention messages and delivery mode.

Additionally, two focus groups (one from each treatment arm) were conducted with sampled Hygienic Family child caregivers. The discussions assessed the acceptability of the intervention messages; delivery mode (use of cluster meetings and household visits), including acceptability towards cues of action; and environmental prompts, e.g., buntings, baby bibs, bracelets, badges, and hand-painted plates.

#### 2.3.5. Household Survey Questionnaire

A structured household survey questionnaire was conducted among hygienic family child caregivers from both treatment group 1 (*n* = 323) and treatment group 2 (*n* = 306) post intervention. Through the household survey, data on basic demographic variables, attendance, and recall of key intervention messages were collected. Additionally, the household survey assessed the presence of promoted hygiene proxy measures, such as handwashing facilities and dish racks.

#### 2.3.6. Expenditure Review

Recorded expenses for each behavior package (*n* = 4) were reviewed to assess how much was spent per package to estimate the intervention implementation cost. The initial costs were in Malawi kwacha, which were converted into U.S. dollars (USD) at the exchange rate of 2018 when the intervention was implemented.

We report the estimated costs of delivering the intervention among 800 households in the treatment area, rural Malawi. Costs for developing the intervention package were not included since they were completed on a separate development process. As such, the costs evaluated in this study relate solely to the training of community coordinators and the delivery of the intervention to the population, which includes all necessary resources to undertake the intervention activities, for example, purchasing paint, Glo Germ^TM^, soap for handwashing, and production and printing of environmental prompts and cues of action (e.g., baby bibs, buntings, and bracelets).

### 2.4. Data Management and Analysis

#### 2.4.1. Quantitative Data

Quantitative data indicating number of activities planned to be implemented (documented in training manuals) were compared against the actual number of activities delivered (field reports) in Microsoft Excel Version 16 (Microsoft corporation, Redmond, WA, USA), where differences were examined to assess fidelity. The attendance of caregivers in cluster meetings and the number of caregivers reached through household visits were extracted from group coordinator reports, cleaned manually, and imported to Statistical Package for Social Sciences (SPSS), version 25.0 (SPSS Inc., Chicago, IL, USA) for analysis. SPSS was used to analyze data from household questionnaires to determine the percentage of caregivers who remembered the key intervention messages and who were observed to have hygiene proxy measures in place.

Linear regression analysis was used to determine if there was any relationship between cluster meeting and household attendance against availability of hygiene proxy measures (WASH infrastructure that were promoted by the Hygienic Family intervention) at the household.

The intervention cost was determined using expenditure reports. All costs associated with the delivery of the intervention, including payments to community coordinators, printing and binding of training materials, purchasing of materials used during cluster meetings and household visits, and awards given to caregivers, contributed to the full cost per household.

#### 2.4.2. Qualitative Data

Qualitative data from FGDs and interviews were voice recorded, transcribed, and thematically analyzed. Transcriptions were read and verified by five research team members before coding for seven identified themes (recruitment, fidelity, reach, dose, acceptability, participant engagement, and participant responses). MS Excel was used to assess data by process evaluation constructs, which also included participants reactions related to the targeted behaviors.

### 2.5. Ethical Considerations

The Hygienic Family intervention had ethical approval from the University of Malawi’s College of Medicine Research Ethics Committee (P.04/16/1935) and was registered with the Pan African Clinical Trial Registry (PACTR201703002084166). All interviewed respondents provided oral informed consent for participation in the study, and no names were mentioned or recorded during the FGDs and interviews.

## 3. Results

### 3.1. Demographic Characteristics

At the end of the intervention, 617 caregivers (representing 617 households) were included in the process evaluation through the structured questionnaire. The child caregivers had an average age of 29 years (min: 17 years; max: 75 years). Fifty percent of the child caregivers earn their living through farming, and 50% of the households earned less than USD 1.50 per day. Over 25% of the child caregivers had never received a formal education (Table 2).

Two FGDs were conducted with community coordinators who were delivering the intervention. The average age of the community coordinators was 29 years old (min: 19 years; max: 41 years), and almost all community coordinators (99%) received secondary education. Additionally, fourteen HSAs and twenty chiefs were included in the process evaluation through FGDs. The age and education level of the HSAs and chiefs was not captured.

### 3.2. Implementation Dose and Reach

By the end of the intervention, the community coordinators received all trainings that were planned pertaining to the delivery of the intervention. However, HSAs (who were to provide a supervisory role) were not trained in one behavior package (water management) due to time constraints and availability.

The process evaluation indicated that all planned activities were delivered by the end of the intervention, giving it a 100% dose rate. Twenty-nine percent of the cluster meetings and eight percent of household visits were rescheduled because of low attendance by the child caregivers and failure of the community coordinator to fulfill their day’s activities. For instance, some activities set for cluster meetings were omitted, and some clusters had challenges in finding a place to hang posters during cluster meetings. In addition, one cluster meeting encountered a technical problem with the public address system that was being used to deliver a video about the importance of handwashing with soap. Nevertheless, these issues were addressed in subsequent cluster meetings, and adequate support was put in place for feedback loops, which meant that challenges could be addressed quickly, and minimized the risk of reoccurrence.

The intervention was designed to reach 800 participants. The end-point evaluation showed a 20% attrition rate. The primary reason for leaving the study was the relocation of participants from the study area due to either marital separation (patrilineal system) or seasonal relocation to farming areas, which were far from the intervention area. The process evaluation did not quantify the percentages for each of the reasons.

Child caregivers were targeted through cluster meetings and household visits, which helped to achieve a good reach of the intervention. Cluster meetings were scheduled once a fortnight, and cluster members chose a day, venue, and time that was most suitable. Household visits were made unannounced once a fortnight, and where a caregiver missed a cluster meeting, community coordinators used the household visit as an opportunity to catch up on what they had missed.

Overall, caregivers from both treatment groups were exposed to the intervention more through household visits than cluster meetings (Table 3), which would be expected due to the one-to-one nature of a visit versus the choice and ability to attend cluster meeting. In most of the behavior packages, more caregivers attended all cluster meetings and household visits more often in treatment group 2 (69%; 84%) than in treatment group 1 (45%; 71%) (Table 3).

The intervention was framed around the Hygienic Family to ensure inclusion of all household members. Therefore, in addition to targeting the child caregivers (female) with the intervention activities, men were also included in specific cluster meetings that pertained to their traditional roles as key decision makers (WASH inclusive) and provision of infrastructure at household level. For example, men were invited to attend cluster meetings where they learned how to construct a dish rack. Process evaluation data showed that 35% of men attended the recommended cluster meetings. Reasons for low attendance included: men felt out of place because the meetings were dominated by women; men were busy with farming, doing business, and working; and some men had no interest in participating in the intervention. The participation of men at household visits was not measured.

*Quote 1: “My husband is a busy person so he cannot manage to attend such meetings.”*

(Caregiver, treatment group 1)

### 3.3. Intervention Fidelity

At the outset, through the community meetings (i.e., open days), caregivers were supposed to make a public commitment that they were part of the Hygienic Family movement. However, due to rainy weather conditions, open days were cut short, meaning these commitments were instead recited at the first cluster meeting. Although not in front of a large group and community leadership, this did show commitment to the other caregivers in the cluster and did not appear to impede participation.

The study established that some community coordinators failed to conduct several activities as planned. Failure of community coordinators to conduct planned activities was particularly common in the first two cluster meetings, corresponding with their relative inexperience in executing their responsibilities. However, with time and supportive supervision, all planned cluster meetings and household visits were delivered as per the protocol. Additionally, community coordinator trainings and supervisory visits were delivered in both treatment groups by the end of the intervention, thus confirming the fidelity of what was delivered.

The intervention planned to distribute prompts and nudges to all study participants in order to support sustained behavior change, i.e., baby bibs, bracelets, and buntings. To facilitate behavior change, local language was used on the prompts; i.e., on a baby bib, it was written “*Kodi mwasamba mmanja ndi sopo*? (Have you washed your hands with soap?)”. The prompts served different purposes as follows:

The baby bibs were meant to prompt handwashing with soap before child feeding and were distributed to the child caregivers during delivery of the food hygiene module. However, fidelity was affected due to late distribution, i.e., baby bibs were distributed later than planned as a result of supply chain challenges, and because the children had grown (from the target age of 6–12 months) up to 18 months, they were rarely used. By the end of the intervention, 85% of the caregivers still had the baby bibs, indicating that they were valued but mostly unused and therefore not serving their intended purpose.

All child caregivers were also provided with rubber bracelets with a message, i.e., “*Ndi masamba mmanja ndi sopo* (I wash my hands with soap)”, which reminded them to wash hands with soap at the targeted critical times. By the end of the intervention, 56% of the caregivers were still found wearing the bracelets. Although it was reported that the messages on the bracelets faded away quickly, the bracelets did provide both a level of prestige (being a Hygienic Family) and continued to be a reminder when worn. Among those who no longer had them, they were reported as lost or broken. Additionally, there was an issue in some households as to who was to use bracelets, either the mother (i.e., the primary child caregiver) or the husband to the targeted child, with men often demanding ownership of the bracelet.

### 3.4. Intervention Receipt

We assessed the intervention receipt through examining acceptability, engagement, and responses to the interventions from caregivers, community coordinators, and our other stakeholders.

#### 3.4.1. Caregivers

The key differences between these two groups was the number of behavior packages (treatment 1 received two packages, and treatment 2 received four behavior packages) and the treatment arm coordinators. Treatment group 1 had a male intervention coordinator, while treatment group 2 had a female treatment arm coordinator. Having a male coordinator made some child caregivers uncomfortable. However, we assume that unobserved factors such as influence from community leaders may have also played a role in the attendance of the caregivers.

*Quote 2: “I found it uncomfortable for a male group coordinator to visit and inspect inside my house.”*

(Caregiver, treatment group 1)

Generally, the intervention recipients were highly engaged in the intervention and responded positively as evidenced by the presence of distributed prompts and promoted hygiene proxy measures at the household level post intervention (Table 4).

However, it was clear that there was variation in caregiver participation, and as such, the impact of the number of visits and level of participation was determined through regression. The number of visits/meetings that participants received/attended (number, continuous) was regressed onto the outcome of interested (binary outcome) individually to determine the direction and magnitude of the relationship (Table 5). For the handwashing with soap package, the number of visits/meetings were not significant in predicting any of the four outcomes. For the food safety package, both the number of household visits and cluster meetings were significant in predicting three of the outcomes. The number of household visits for the feces management package were significant in predicting two of the outcomes though the relationship was negative; i.e., an increased number of visits reduced the probability of achieving the outcome.

Caregivers were engaged through a range of “fun” activities, and they expressed particular interest in the activities conducted during practical sessions. For example, a number of participants recalled the use of Glo Germ^TM^ (Moab, UT, USA) (an interactive visual aide for hygiene training) via fluorescent hand lotion, where they could see the “germs” on their hands, which they could not see with their naked eyes, and they could appreciate the effectiveness of handwashing with soap in removing “germs”.

*Quote 3: “One of the activities that I liked was the Glo Germ^TM^ activity. The Glo Germ^TM^ activity helped me see how handwashing with soap is effective to get rid of germs on our hands. This activity always reminded me of the importance of handwashing with soap.”*

(Caregiver, treatment group 2)

This may have contributed to the message recall assessed during the end-line household survey, which showed that 100% of caregivers remembered critical times for handwashing, the importance of handwashing with soap, the importance of food safety and hygiene, and water management in the prevention of diarrheal disease.

The Chikwawa district is among the poorest in Malawi and is prone to flooding in the rainy season; many residents are reliant on relief and incentives to support their day-to-day needs. Although the caregivers eventually accepted the delivery approach of the Hygienic Family intervention, acceptability was difficult at the beginning of the intervention due to a lack of incentives. However, awards were provided to households who performed well as part of the behavior change technique (BCT) of the RANAS behavior change model. As landmarks were achieved, and items were received, caregivers were increasingly motivated and encouraged to practice the promoted hygiene behaviors. For example, households who had functioning handwashing facilities and constructed a raised area for food and utensil storage were awarded with a bar of soap through a public celebration, thus providing both household items and prestige.

#### 3.4.2. Community Coordinators

Community coordinators were financially compensated for the time spent on delivering the intervention. Through FGDs, the process evaluation found that the compensation was accepted among the community coordinators.

*Quote 4: “Before the Hygienic Family, I was solely depending on my husband for survival whom at times he would say he does not have money for some of my needs. But after being given the role to coordinate the mothers on this program, I have been able to take care of myself and my children with the money I get from the program.”*

(Community coordinator, treatment group 2)

Additionally, community coordinators felt respected in the community because they were given that responsibility.

*Quote 5: “Given such a responsibility by the Hygienic Family project has made people to respect me in my community. I remember one teacher approached me and asked me to give a career talk to girls in our community”*

(Community coordinator, treatment group 1)

Community coordinators liked the approach used by the Hygienic Family; however, they found that the unannounced household visits were a challenge. Sometimes, community coordinators would make several attempts to find the caregiver at the household, thus affecting their work plan.

*Quote 6: “Since we were making unannounced visits to the household, it was hectic and overwhelming for me to go to a household several times if I did not find the caregiver at the initial scheduled time.”*

(Community coordinator, treatment 2)

#### 3.4.3. Other Community Stakeholders

In order to develop and maintain the sustainability of the intervention, it was also important for the intervention to engage community-based stakeholders in this program. Feedback through FGDs with chiefs and HSAs were integral to this.

Chiefs supported the intervention by helping community coordinators to mobilize women for cluster meetings.

*Quote 7: “Some women were reluctant to attend cluster meetings, so I used the village meetings as an opportunity to encourage the recruited households to attend meetings.”*

(Chief, treatment group 2)

Additionally, chiefs were impressed with the outcome of the Hygienic Family because of the spillover effect of the intervention.

*Quote 8: “This project selected and recruited few houses in my area but one day when I was walking around, I noticed that other households, which are not in the project have also constructed things like handwashing facilities at their households.”*

(Chief, treatment group 2)

HSAs were also engaged in the intervention as evidenced by their joint facilitation of cluster meetings and household visits. However, some community coordinators reported that they had inadequate support from their respective HSAs. HSAs stated during FGDs that the inadequate support was due to their busy work schedules.

*Quote 9: “Most of the time I was not able to support the community coordinator of my area when conducting cluster meetings and household visits because of my busy work schedule.”*

(HSA, treatment group 1)

However, HSAs recognized and appreciated the intervention.

*Quote 10: “Over time, we have had problems to ensure and sustain good WASH practices among the community members. But with the coming in of the project, we have observed an increase in good WASH practices and reduced diarrhea cases in our village clinics.”*

(HSA, treatment group 2)

### 3.5. Intervention Costs

Having implemented the intervention for 32 weeks in 40 villages (800 households), the total cost was USD 19,877, or an estimated cost of USD 31.00 per household (Table 6). More details on the materials and cost per package can be found in Appendix A.

Due to the long implementation period, the food hygiene package (15 weeks) had the highest cost at USD 12.94 per household followed by USD 8.99 per household for the handwashing with soap package (7 weeks). The feces management and water management packages had the lowest costs due to the short implementation period (four weeks for feces management package and three weeks for water management package). Additionally, on some occasions, the trainers used training materials that had already been used in the previous packages (e.g., paint and illustrations). By the end of the intervention, the intervention team used 57% of the total cost on the intervention implementation materials (USD 11,351).

The training manuals used in the delivery of the intervention package are available on https://doi.org/10.17868/76319 (accessed on 11 March 2021).

## 4. Discussion

In this process evaluation, we described and measured the implementation of a community-based WASH and food hygiene intervention that used the RANAS behavior change model and resulted in a 13-percentage-point reduction in self-reported diarrhea among children aged below five years [17,21]. This evaluation examined a range of factors associated with the intervention implementation and examined the barriers and opportunities available for scaling up this intervention to a wider population.

### 4.1. Implementation Fidelity

Flexibility of timing contributes to the successful delivery of an intervention [22]. In our intervention, some cluster meetings and household visits were rescheduled due to low attendance by the caregivers. This contributed to the delivery of all intervention activities within the project implementation period. This emphasizes the need to ensure that community health programs are flexible in terms of scheduling activities and use of female community coordinators who can be empathetic to other household and family demands. Such flexibility could extend to those delivering the intervention. Concerns from child caregivers about household visits made by male treatment arm coordinators concurs with a study in South Africa that reported that some women preferred a female community health worker than a male community health worker [23]. Thus, use of female community coordinators in such interventions is encouraged, especially where household inspection is part of the process. Identification of context-specific issues, which may arise as a result of facilitator gender, should be examined at the formative stages of intervention development to ensure that they are adequately considered.

The quality of the intervention delivery during the initial stages (i.e., first two cluster meetings of handwashing with soap module) suffered because the community coordinators were unfamiliar with the behavior-centered approach used. However, with time and support from the intervention team, the community coordinators gained skills and confidence, which resulted in improved quality. Hence, as reported elsewhere, provision of supportive supervision to community health program implementers is vital to successful results particularly in the initial stages of implementation [24].

Practical sessions in the delivery of community health interventions have been proven to be helpful elsewhere [25], and similarly, the use of practical demonstrations in the delivery of the Hygienic Family not only improved the child caregivers’ knowledge but also provided hands-on experience with the promoted behaviors. This combined teaching and practical approach is also cognizant of the low level of literacy across the majority of caregivers. As much as environmental prompts and cues for action have been successful in changing behaviors [26], these created challenges in some areas of the intervention. In particular, baby bibs were less effective in promoting the targeted hygiene behaviors. Careful consideration needs to be given to the inclusion of appropriate nudges and cues for action taking into consideration the need for nudges, timing, and the context. Bibs could be retested in this population if introduced at an earlier age and with an appropriate size. Buntings, bracelets, and the hand-printed paper plates fitted well with the intervention, and they should be promoted in similar future interventions. However, researchers need to understand the context first (including power dynamics within the household) before providing personal items such as the bracelets.

Active participation of men in community health interventions require their involvement in all the activities throughout the project cycle in a patriarchal society where decisions are mostly made by men [27]. Although our intervention was meant to focus on all the household members, men were less involved than their female counterparts. Importantly, it is necessary to assess if it is realistic to expect men to participate fully in such health projects, and more effort should be channeled into how these programs can be designed to effectively involve men.

Although it was planned that the community health workers (i.e., HSAs) should be fully participating in conducting cluster meetings and household visits, interviews with the HSAs found that their participation was minimal because they were also responsible for other community health programs, which also required their time. Considering the busy schedule of the HSAs, it is important that the food hygiene interventions promoted through the Hygienic Family be integrated into HSA’s existing work activities rather than being implemented independently. It is possible that full participation of the HSAs could have significant impact on household uptake of the promoted behaviors since they are a trusted source of health information in the communities [28]. Importantly, use of local existing structures is integral in the sustained success of community health programs [29]. As such, the interventions could be delivered through existing community structures (e.g., Village Health Committee and Care Groups) where the HSAs would play a supervisory role to the structures.

### 4.2. Intervention Reach and Receipt

Intervention attrition was primarily due to migration and seasonal relocation of caregivers. Consistent participation is integral to achieving behavior change [30], and as such, it is important that community involvement and social and intervention mapping exercises are conducted at the onset of a WASH (including food hygiene) intervention to ensure that reach of the intervention is maximized [31,32,33]. For instance, in the case of this study, a touch-point-mapping exercise could have indicated that there would be a need to reach out to some child caregivers at their seasonal homes or ensure that the content was covered during their stay at their village home; this should be incorporated into the design of future interventions.

Attendance across both cluster and household visits was intermittent, which was reflective of the need for caregivers to address day-to-day household needs. From this perspective, household visits were important to reach out to those child caregivers who missed the cluster meetings and provided one-to-one interactions to both reinforce and reiterate messages. This was also supported by repetition of content across modules, ensuring messages were received by everyone and supporting the time needed to actualize behavior change [34]. Additionally, the food safety package, which had a longer delivery than the other behavior packages, significantly predicted three out of the four of the outcomes through the use of both household visits and cluster meetings. Thus, use of both cluster meetings and household visits support behavior change when delivered for an adequate period. Additionally, delivering a sufficient dose of an intervention is essential for behavior change [35].

Generally, all stakeholders were satisfied with the approach used in the Hygienic Family intervention, evidenced by continued participation in intervention activities. The use of emotive motivators such as Glo Germ^TM^ provided clear, visible, and practical evidence of handwashing with soap efficacy and, as with previous studies, emphasizes the importance of visualization of risk to increase understanding and the need for action [26].

### 4.3. Cost

The intervention implementation cost USD 31.00 per household. This could infer that the cost of scale is prohibitive within a LMIC economy. However, the cost could be further reduced by using alternatives for some materials such as paint instead of Glo Germ^TM^ to demonstrate the effectiveness of soap in removing germs and reducing the number of awards given to caregivers for meeting intervention landmarks. This intervention achieved a significant reduction in diarrheal disease [17], and this should be taken into consideration with the cost, as it has been estimated that an episode of diarrhea can cost a household between USD 1.81 and USD 19.16 for outpatient and inpatient treatment, respectively [36].

A significant proportion of the cost of the intervention also included costs for stipends that were given to the community coordinators every month to help them support their families. Compensating coordinators for their time motivated them to work hard and complete assigned activities. This concurs with a study that found that not compensating coordinators affected how they do their work as a result of being disrespected by other community members and opting to do “piece work” to earn money to support their families rather than doing project work that they were not being paid for [37]. There are additional benefits to providing financial compensation for community coordinators, providing them not only with security to undertake their role but also providing them with prestige in the community [16]. The costs for paying community coordinators could be reduced if the interventions are delivered by village health committee (VHC) members. However, this may affect the fidelity and reach of the intervention. Considering the socio-economic status of most people in such a setting, it is challenging to expect someone to spend more time doing something on a voluntary basis when there are opportunities for income generation in other areas.

### 4.4. Limitations

Firstly, we did not measure male participation at household visits and are therefore unable to provide strategies to motivate male participation at this time but recommend this as integral to future formative work. Secondly, the study was done only in one district, which is not necessarily representative of the whole region or country. Additionally, the study used the number of attendees to measure reach of the intervention. Other studies have found that attendance does not necessarily mean reach because not everyone would internalize the message. However, we feel that reach can be related to attendance in our study because the intervention was hands-on, ensuring full participation.

## 5. Conclusions

The process evaluation showed that the Hygienic Family intervention achieved good fidelity, dose, reach, and acceptability. Using existing structures in the communities such as caregiver groups and community health workers (HSAs) have been shown to be effective in the delivery of this WASH and food hygiene intervention. However, this process evaluation has also shown that this needs to be reinforced with significant support and supervision of intervention mechanisms, ideally with financial compensation for key personnel at community level who would traditionally be recruited as volunteers. This provides the necessary sustainable structure while building social capital and prestige for individuals and community members and could be used to widen the scope of content for child health and wellbeing. The intervention design was based on formative work, which took into consideration the need for alternative routes of communication, practical application, and the distractions of day-to-day life.

The results of this process evaluation suggest that the Hygienic Family intervention approach could be used as an example for interventions in similar settings in sub-Saharan Africa and beyond. However, given the context specific nature of the intervention, planners should be careful in generalizing the results of the planning process to other populations.

## Figures and Tables

**Table 1:** Overview of Process Evaluation methods.

Search Method or Data Source	Total Number of Data Sources Used		Data Type	Respondents	Core Information Sought	Purpose of Information	Timing
	Treatment 1	Treatment 2 *					
Implementation summary reports	26	34	Quantitative	Treatment arm coordinators	Activities conducted during cluster meetings and household visits and challenges faced. Number of cluster meetings and household visits conducted. Number of men and women who attended cluster meetings and were visited in their households (household visits).	Fidelity, dose delivered, and reach	Throughout the intervention
Weekly activity reports	26	34	Qualitative	Community coordinators	Content and quality of delivery and participant engagement. Contextual information on delivery and receipt in clusters and household visits. Reasons for any deviations from planned activities and adaptations made	Fidelity, participant engagement, and context	Throughout the intervention
In-depth interviews	3		Qualitative	Community coordinators, treatment arm coordinators, and intervention coordinator)	Perspective of the implementers on the successes and challenges of intervention delivery. Recruitment strategies and challenges across the clusters and intervention areas. Acceptability of the intervention messages, delivery mode, and activities	Fidelity, recruitment, context, acceptability, and participant engagement and responses	Post intervention
Community coordinator training reports	4		Quantitative	Intervention coordinator and treatment arm coordinators	Activities conducted during training. Number of trainings offered to community coordinators and number of community coordinators who attended each behavior package training	Fidelity, dose delivered, and reach	Throughout the intervention
Supervisory visits	6		Quantitative	Intervention lead and research fellow	Content and quality of delivery and participant engagement. Contextual information on delivery and receipt in clusters and household visits	Fidelity, participant engagement, and context	Throughout the intervention
Focus group discussions conducted	3	3	Qualitative	Health surveillance assistants, chiefs, and community coordinators	Perspective on the successes and challenges of intervention delivery. Recruitment strategies and challenges across the clusters and intervention areas. Acceptability of the intervention messages, delivery mode, and activities	Fidelity, recruitment, context, acceptability, and participant engagement and responses	Post intervention
	1	1	Qualitative	Sample of individuals in intervention arms	Acceptability of the intervention messages, delivery mode, and activities including acceptability towards cues of action and environmental prompts, e.g., buntings, baby bibs, bracelets, badges, and hand-painted plates.	Acceptability	Post intervention
Household survey questionnaire	323	306	Quantitative	Individuals in intervention arms	Proportion of individuals reporting attendance of each intervention component. Recall and recognition of intervention concept and messages. Presence and use of promoted infrastructure for hygiene.	Fidelity, reach, and acceptability	Post intervention
Expenditure review	4		Quantitative	Intervention expenditure reports (per behavior package)	How much money was spent to implement the intervention	Estimating cost of intervention	Throughout the intervention

* additional reports due to longer implementation (8 weeks) in Treatment Group 2.

**Table 2:** Demographic characteristics of the child caregivers (*n* = 617).

		Number of Care Givers (*n* = 617) (Count (%))
Age	Mean	29 (17–75 years)
Marital status	Married	564 (88.8%)
	Divorced	39 (6.1%)
	Single	20 (3.1%)
	Widow	12 (2%)
Education level	Never been to school	158 (24.9%)
	Primary school	423 (66.6%)
	Secondary school	49 (7.7%)
	Tertiary education	5 (0.8%)
Occupation	Farming	310 (48.8%)
	Business	180 (28.3%)
	Employed	25 (4.0%)
	Housewife	26 (4.1%)
	Others	94 (14.8%)
Average income per month	USD 0.00–USD 13.20	141 (22.2%)
	USD 13.29–USD 26.50	116 (18.3%)
	USD 26.58–USD 39.80	63 (9.90%)
	USD 39.87–USD 53.10	28 (4.40%)
	USD 53.16–USD 66.40	252 (39.7%)
	>Over USD 66.45	35 (5.50%)

**Table 3:** Caregivers’ attendance at cluster meetings and household visits.

Delivery Method	Package	Treatment Group 1									Treatment Group 2								
		Percentage of Attendance									Percentage of Attendance								
		0	1	2	3	4	5	6	7	8	0	1	2	3	4	5	6	7	8
Cluster meeting attendance by child caregivers	Handwashing with soap (*n* = 4 meetings)	9%	4%	21%	27%	39%					17%	4%	10%	28%	42%				
	Food safety and hygiene (*n* = 8 meetings)	27%	1%	2%	3%	8%	15%	19%	11%	14%	5%	3%	6%	6%	10%	12%	16%	22%	19%
	Feces management (*n* = 3 meetings)										2%	1%	9%	87%					
	Water management (*n* = 2 meetings)										5%	20%	76%						
Household visit attendance by child caregivers	Handwashing with soap (*n* = 3 visits)	4%	32%	14%	50%						4%	1%	10%	85%					
	Food safety and hygiene (*n* = 7 visits)	3%	1%	1%	2%	5%	10%	38%	39%		4%	1%	1%	1%	3%	10%	39%	42%	42%
	Feces management (*n* = 3 visits)										4%	17%	79%						
	Water management (*n* = 1 visit)										9%	13%	78%						

Note: Shaded parts implies that the behavior package did not include that number of cluster or household visit. Treatment group 1 did not receive the intervention packages related to feces management and water management.

**Table 4:** Proxy measures compared between treatment 1 and treatment 2 at baseline (2017) and end line (2018)) Adapted with permission from [17]. ©2020, Morse T.

	Treatment 1		Treatment 2	
Proxy Measures	Baseline (*n* = 400)	End Line (*n* = 323)	Baseline (*n* = 400)	End Line (*n* = 306)
Presence of soap at HH	61% (243)	93% (302)	59% (234)	93% (285)
Presence of handwashing facility (HWF)	41% (164)	98% (316)	44% (176)	95% (291)
Presence of soap and water at HWF	21% (84)	84% (271)	9% (36)	70% (213)
Presence of soap and water at utensil washing location	33% (132)	72% (231)	32% (128)	68% (208)
Presence of dish rack	27% (108)	98% (316)	30% (120)	97% (298)

**Table 5:** Influence of cluster meetings and household visits on the presence of the hygiene proxy measures in the households.

Hygiene Proxy Measure	Handwashing w/Soap Package		Food Safety Package		Water Management Package		Feces Management Package	
	Household Visits *β (SE)*	Cluster Meetings *β (SE)*	Household Visits *β (SE)*	Cluster Meetings *β (SE)*	Household Visits *β (SE)*	Cluster Meetings *β (SE)*	Household Visits *β (SE)*	Cluster Meetings *β (SE)*
Presence of soap @HH (1/0)	0.004 (0.013)	0.011 (0.008)	0.003 (0.011)	0.003 (0.004)	0.027 (0.040)	0.037 (0.032)	−0.060 (0.043)	−0.011 (0.034)
Presence of dish rack @HH (1/0)	0.002 (0.012)	0.011 (0.007)	**0.018** * (0.010)	**0.007** * (0.004)	−0.057 (0.039)	**0.054** * (0.031)	**−0.081** * (0.042)	0.053 (0.033)
Presence of handwashing facility @HH (1/0)	0.001 (0.010)	0.005 (0.006)	**0.022** ** (0.008)	**0.010** *** (0.003)	−0.022 * (0.036)	0.027 (0.029)	**−0.067** * (0.038)	0.017 (0.030)
Presence of soap at handwashing facility (1/0)	0.001 (0.021)	0.013 (0.013)	**0.039** ** (0.017)	**0.014** ** (0.007)	−0.084 (0.068)	0.010 (0.054)	−0.065 (0.071)	0.023 (0.057)

*: *p*-value < 0.1. **: *p*-value < 0.05. ***: *p*-value < 0.01.

**Table 6:** Overall estimated delivery cost for 36-week Hygienic Family intervention (US dollars).

Behavior Package	Cost per Household (USD)	Cost per Cluster (USD)	Cost per Package (USD)
Handwashing with soap package (Households *n* = 800; clusters *n* = 40) (4HH visits) (5 cluster meetings)	8.99	171.82	6242.84
Food hygiene package (Households *n* = 800; clusters *n* = 40) (8 HH visits) (8 cluster meetings)	12.94	258.6	10,043.66
Feces management package (Households *n* = 400; clusters *n* = 20) (3 HH visits) (4 cluster meetings)	4.77	95.59	1911.87
Water management package (Households *n* = 400; clusters *n* = 20) (3 HH visits) (3 cluster meetings)	4.2	83.93	1678.65
Total (USD)	30.9	609.94	19,877.02

## Data Availability

All data referred to within this study is available upon request.

## Appendix A

**Table A1:** Overall Estimated Intervention Delivery Cost (US Dollars).

Behavior Package Materials	Cost per Behavior Package (USD)	Cost per Cluster (USD)	Cost per Household (USD)
Handwashing with soap package (Households *n* = 800; clusters *n* = 40)			
Stationery	1065.23	26.63	1.33
Hygiene consumables	115.60	2.89	0.14
Field equipment	169.80	4.25	0.21
Awards	111.55	2.78	0.14
Implementation activity materials	2702.35	67.56	3.78
Personnel	2078.31	67.71	3.39
Subtotal	6242.84	171.82	8.99
Food hygiene package (Households *n* = 800; clusters *n* = 40)			
Stationery	489.22	19.73	0.99
Hygiene consumables	254.24	6.36	0.32
Field equipment	169.49	4.24	0.21
Awards	574.28	14.36	0.72
Implementation activity materials	4814.06	120.35	6.02
Personnel	3742.37	93.56	4.68
Subtotal	10,043.66	258.60	12.94
Feces management package (Households *n* = 400; clusters *n* = 20)			
Stationery	27.12	1.36	0.07
Hygiene consumables	13.56	0.68	0.03
Field equipment	101.69	5.08	0.25
Awards	162.72	8.14	0.41
Implementation activity materials	569.49	28.47	1.42
Personnel	1037.29	51.86	2.59
Subtotal	1911.87	95.59	4.77
Water management package (Households *n* = 400; clusters *n* = 20)			
Stationery	10.85	0.54	0.03
Hygiene consumables	0.00	0.00	0.00
Field equipment	0.00	0.00	0.00
Awards	0.00	0.00	0.00
Implementation activity materials	0.00	0.00	0.00
Personnel	1667.80	83.39	4.17
Subtotal	1678.65	83.93	4.20
Grand total (USD)	**19,877.02**	**609.94**	**30.9**
